# Abnormal bleeding after lumbar vertebrae surgery because of acquired factor XIII deficiency: A case report and literature review

**DOI:** 10.1097/MD.0000000000036944

**Published:** 2024-01-12

**Authors:** Peng Zhang, Ruijing Zhang, Cheng Jing

**Affiliations:** a Shandong University of Traditional Chinese Medicine, Jinan, Shandong, China; b Beijing University of Traditional Chinese Medicine, Beijing, China; c The Affiliated Hospital of Shandong University of Traditional Chinese Medicine, Jinan, Shandong, China.

**Keywords:** coagulopathy, factor XIII deficiency, hypofibrinogenemic, lumbar vertebrae

## Abstract

**Rationale:**

Abnormal bleeding due to low fibrinogen (Fib) and coagulation factor XIII (FXIII) levels after lumbar vertebral surgery is exceedingly rare. Excessive bleeding is also associated with secondary hyperfibrinolysis. This report presents a case of abnormal incision bleeding caused by coagulation factor XIII deficiency (FXIIID) and secondary hyperfibrinolysis in a state of low fibrinogen after lumbar vertebral surgery.

**Patient concerns:**

A middle-aged woman experienced prolonged incision and excessive bleeding after lumbar vertebral surgery.

**Diagnosis:**

Combined with coagulation factors, coagulation function tests, and thromboelastography, the patient clinical presentation supported the diagnosis of FXIIID and secondary hyperfibrinolysis in a hypofibrinogenemic state.

**Interventions:**

Cryoprecipitat, Fresh Frozen Plasma, Fibrinogen Concentrate, Leukocyte-depleted Red Blood Cells, Hemostatic (Carbazochrome Sodium Sulfonate; Hemocoagulase Bothrops Atrox for Injection; Tranexamic Acid).

**Outcomes:**

After approximately a month of replacement therapy and symptom treatment, the patient coagulation function significantly improved, and the incision healed without any hemorrhage during follow-up.

**Lessons:**

Abnormal postoperative bleeding may indicate coagulation and fibrinolysis disorders that require a full set of coagulation tests, particularly coagulation factors. Given the current lack of a comprehensive approach to detect coagulation and fibrinolysis functions, a more comprehensive understanding of hematology is imperative. The current treatment for FXIIID involves replacement therapy, which requires supplementation with both Fib and FXIII to achieve effective hemostasis.

## 1. Introduction

The lumbar vertebrae are susceptible to motor and neurological dysfunction in chronic injury and/or acute trauma because of their momentous physiological and anatomical functions and biomechanics. Surgical procedures are selected when severe symptoms are present and postoperative complications are common. Hemorrhage, the most common postoperative complication, generally recovers with normal incision healing. An abnormally prolonged duration of bleeding may be associated with infection, poor wound healing, vascular injury, coagulation, and fibrinolytic dysfunction. Coagulation and fibrinolysis are complex physiological and pathological mechanisms that require the participation of numerous components such as fibrinogen (Fib), coagulation factors, red blood cells, and platelets (PLT). Unlike other coagulation factors, coagulation factor XIII (FXIII), also known as the “fibrin stabilizing factor,” increases the resistance of blood clots to mechanical stress and fibrinolysis. Therefore, patients with coagulation factor XIII deficiency (FXIIID) may present abnormally prolonged bleeding.

In this report, we describe a middle-aged female patient with persistent incisional bleeding following lumbar vertebrae surgery due to acquired FXIIID and secondary hyperfibrinolysis in a low-fibrinogen state and review the literature related to coagulation and fibrinolysis.

## 2. Case presentation

The subjects of this study were middle-aged women who had previously exhibited good physical health characterized by heavy menstrual volume and regular menstrual cycles. The patient and her family denied any clinical history of significant trauma, substance abuse, or family history of hematologic disease. She had been transfused for blood loss during cesarean section more than 20 years ago and had undergone surgery for bilateral varicose veins of the lower extremities 10 years ago.

On February 9, 2023, he was admitted to a local hospital with numbness and pain in the left lower limb. Routine coagulation tests on admission revealed that the plasma levels of Fib was 1.24 g/L, D-dimer (D-D) was 2.83 µg/ml, red blood cells (RBC) was 3.47 × 10^12/L, hemoglobin (HGB) was 96 g/L and PLT was 143 × 10^9/L. The patient received a supplemental suspension of leukocyte-depleted red blood cells (LPRC) and fresh frozen plasma (FFP), with a preoperative RBC of 3.47 × 10^12/L, HGB of 113 g/L, and PLT of 161 × 10^9/L. The surgical procedure was performed on March 9 at a local hospital with approximately 300 ml of intraoperative blood loss. The amount of bleeding was over 1000 ml and the total amount of LPRC and FFP transfused was approximately 1600 ml before being transferred to the Department of Microtrauma Orthopedic Surgery, Shandong Orthopedic Hospital.

**Figure 1.**
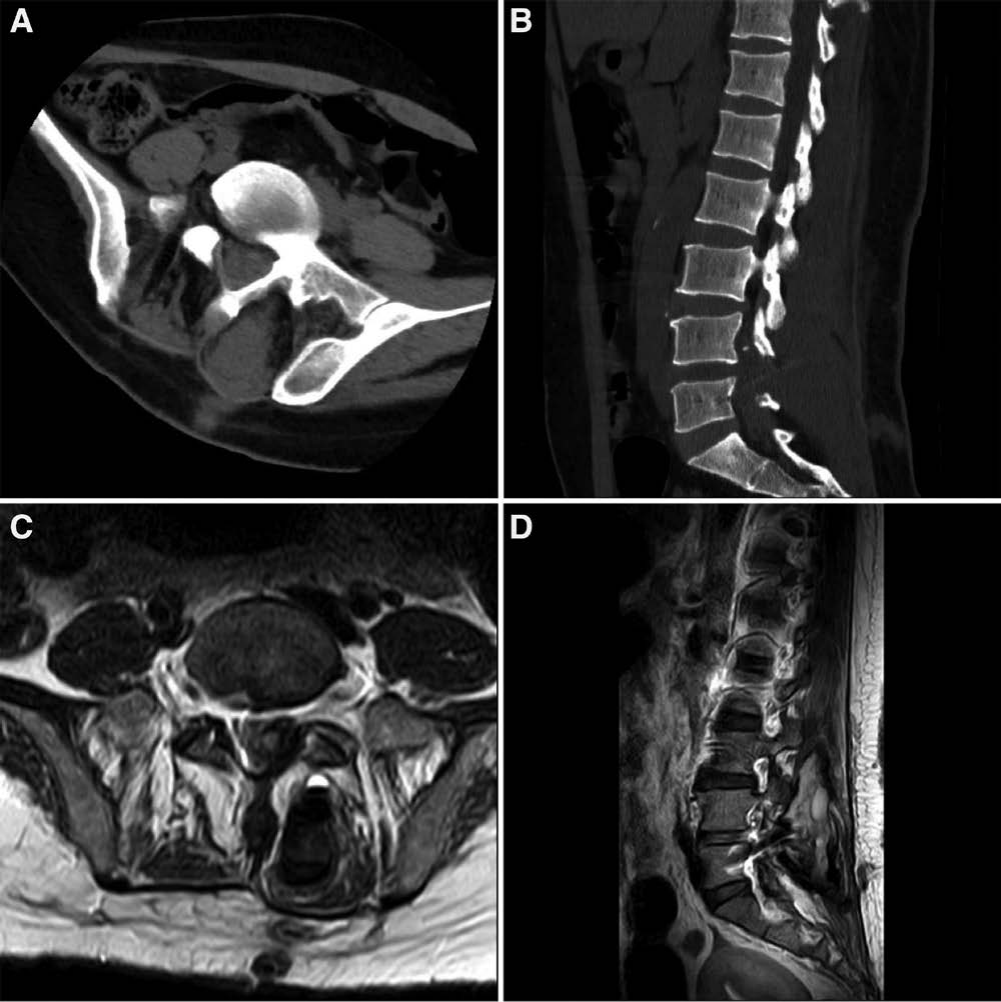
CT (A, B) and MRI (C, D) of the lumbar vertebrae showed the possibility of multiple hematomas within the vertebral canal and in the left posterior aspect of the vertebral body and subcutaneous adipose tissue.

On admission, the incision was covered with sterile dressings soaked in blood; however, it was well closed at the margin of the skin with a small amount of scab, and there was no apparent fever, redness, swelling, or subcutaneous hemorrhage. Tranexamic acid (TXA, 1.25 g) for coagulation and sodium atescinate (10 mg) for anti-inflammatory and anti-exudative effects, improving circulation, and protecting blood vascular walls were empirically administered before the examination. Laboratory tests revealed normal hepatic and renal functions, abnormal hemorrhagic anemia, and coagulation (Table 1). CT and MRI of the lumbar vertebrae showed the possibility of multiple hematomas within the vertebral canal, in the left posterior aspect of the vertebral body, and in subcutaneous adipose tissue (Fig. 1). On March 12, human serum albumin (10 g) and FFP (200 ml) were confused due to low albumin, fibrinogen, and anemia; On March 13, tests for coagulation factors indicated a decrease in Fib (critical value), FXIII, and HG (Table 1), and LPRC (4U), FFP (400 ml), and cryoprecipitate (Cryo, 10U) were administered; On March 14, in the condition of the critical value of Fib, Cryo (12U) was administered; On March 15, the level of Fib was further reduced and fibrinogen concentrate (FC, 2 g) was given to correct the critical value after consulting a hematologist; On March 16, the Fib level rebounded and human FC (1 g) was continued (Tables 2 and 3). During these 5 days, the amount of blood oozing from the incision decreased. A full set of coagulation tests (Table 1) revealed extremely high levels of fibrinogen degradation products (FDP, > 150µg/ml), elevated soluble fibrin monomers (FM, 91.99µg/ml), and D-D (60.00µg/ml). On March 19, the critical fibrinogen value was observed again, and FC (1 g) was infused. In combination with previous coagulation tests, this status was thought to impair fibrin stabilization due to FXIIID in the low-fibrinogen state, which was associated with secondary hyperfibrinolysis.

**Table 1:** Routine laboratory investigations including liver and kidney function, electrolytes, blood routine, routine coagulation function on March 12, 2023; coagulation factor on March 13, 2023; complete set of coagulation function on March 17, 2023.

Time	Examination		Result	Reference range	Unit
2023/3/12	Liver function	ALT	8	7–40	U/L
		AST	21	13–35	U/L
		TBil	18.6	0–21	μmol/L
		DBil	5.3	0–4	μmol/L
		IBiL	13.3	0–17	μmol/L
		TP	48.1	65.0–85.0	g/L
		ALB	25.8	40.0–55.0	g/L
		PA	74	180–350	mg/L
		ALP	26	35–100	U/L
		γ-GGT	13	7–45	U/L
		ADA	16.2	0–25.0	U/L
		AFU	15	0–40	U/L
2023/3/12	Kidney function	BUN	2.20	2.6–7.5	mmol/L
		Cr	35	41–73	μmol/L
		UA	167	155–357	μmol/L
		β2-MG	1.8	1.0–2.3	mg/L
		GFR	127.59	>90	Ml/min
2023/3/12	Electrolytes	Ca	1.86	2.11–2.52	mmol/L
		Na	137	137–147	mmol/L
		K	3.27	3.50–5.30	mmol/L
		Cl	111	99–110	mmol/L
2023/3/12	Blood routine	WBC	4.96	3.5–9.5	10^9/L
		RBC	2.86	3.80–5.10	10^12/L
		HGB	80	115–150	g/L
		PLT	89	125–350	10^9/L
		HS-CRP	12.90	0.00–10.00	mg/L
2023/3/12	Routine coagulation function	PT	15.5	11.0–15.0	S
		INR	1.20	0.80–1.20	INR
		APTT	37.7	28.0–43.5	S
		Fib	1.12	2.00–4.00	g/L
		TT	21.1	14.0–21.0	S
		D-D	5.07	0–0.50	μg/ml
2023/3/13	Coagulation factor	Factor II	73.8	50~150.	%
		Factor V	73.6	50~150.	%
		Factor VII	108.6	50~150.	%
		Factor VIII	80.2	50~150.	%
		Factor IX	84.7	50~150.	%
		Factor X	83.9	50~150.	%
		Factor XI	84.2	50~150.	%
		Factor XII	39.9	50~150.	%
		Factor XIII	20.2	50~150.	%
		VWF:Ag	264.7	66.1~176.3/AB	%
2023/3/17	Complete set of coagulation function	TT	16.1	11.0–15.0	S
		INR	1.28	0.80–1.20	INR
		APTT	39.7	28.0–43.5	S
		Fib	1.07	2.00–4.00	g/L
		TT	20.5	14.0–21.0	S
		D-D	60.00	0–0.50	μmol/L
		FDP	>150	0–5	μmol/L
		AT-III	89	80–120	%
		PC	88.00	70–130	%
		VWF:Ag	420.00	50–160	%
		FM	91.99	0.1–6	μmol/L
		LA-S	1.12	≤1.2	
		LA-S R	38.20		S

ADA = adenosine deaminase, AFU = a-L-fucosidase, ALB = albumin, ALP = alkaline phosphatase, ALT = alanine aminotransferase, APTT = activated partial thromboplastin time, AST = aspartate aminotransferase, AT-III = pplasma antithrombin III activity, BUN = blood urea nitrogen, Cr = creatinine, DBiL = direct bilirubin, D-D = D-dimer, FDP = fibrinogen degradation products, FIB = fibrinogen, FM = soluble fibrin monomer, GFR = glomerular filtration rate, HGB = hemoglobin, HS-CRP = high-sensitivity C-reactive protein, IBiL = indirect bilirubin, INR = international normalized ratio, LA-S = lupus anticoagulant screening, LA-S R = lupus anticoagulant screening rate, PA = prealbumin, PC = plasma protein C activity, PLT = platelets, PT = plasma thrombin time, PT = prothrombin time, RBC = red blood cells, TBiL = total bilirubin, TP = total protein, TT = thrombin time, UA = uric acid, vWF = von willebrand factor, WBC = white blood cells, β2-MG = β2-microglobulin, γ-GGT = γ-glutamyltransferase.

**Table 2:** The results of thromboelastography.

	R	K	Angle	MA	CI	EPL	LY-30
Reference range/Unit	4–8 min	1–3 min	53–72 deg	50–70 mm	~3~3	0–15%	0–7.5%
2023/3/12	5.4	3.5	51.7	46	−3.1	0	0
2023/3/17	6.8	6.8	33.6	38.2	−7.6	0	0
2023/4/3	4.8	3.2	52.6	48.7	−2.2	0.1	0.1

**Table 3:** Coagulation function tests during hospitalization.

	HGB	PLT	INR	PT	APTT	Fib	TT	D-D	FDP
Reference range/Unit	115–150g/L	125–350 × 10^9/L	0.8–1.2INR	11.0–15.0s	28.0–43.5s	2.00–4.00g/L	14.0–21.0s	0–0.50μmol/L	0–5μmol/L
2023/2/9	/	/	1.18	14.2	32.4	1.24	18.6	2.83	/
2023/3/11	82	120	1.37	16.6	39.4	1.36	17.8	/	/
2023/3/12	80	89	1.2	15.5	37.7	1.12	21.1	5.07	/
2023/3/13	87	93	1.24	15.9	40.7	0.79	26.4	5.73	/
2023/3/14	97	84	1.22	15.7	35.2	0.83	25	2.32	/
2023/3/15	96	99	1.49	18.4	39	0.61	30.1	>20	/
2023/3/16	101	103	1.32	16.7	38.8	1.06	21.6	>20	/
2023/3/17	107	93	1.28	16.1	39.7	1.07	20.5	60	>150
2023/3/19	/	/	1.28	16.3	41.9	0.89	22.6	1.55	/
2023/3/20	/	/	1.3	16.4	39.9	0.96	22.9	>20	/
2023/3/24	94	100	1.34	16.8	42.2	0.93	23.1	>20	/
2023/4/1	88	119	1.4	17	41.3	0.92	20.3	>20	>150
2023/4/3	88	119	1.17	14.9	38.9	1.72	18.80	>20	>150

Owing to technical constraints, the patient was transferred to a higher-level medical center, the Hematology Department of Qilu Hospital of Shandong University, on March 24 for follow-up treatment, which was reasonably adjusted based on the patient test results: Cryo (16U Qd), FFP (200 ml Qd), FC (1 g Bid), Carbazochrome Sodium Sulfonate (80 mg Qd), and Hemocoagulase Bothrops Atrox for Injection (2units Qd). On April 4, the patient was discharged from the hospital with no apparent blood seepage from the incision and an obvious improvement in coagulation function (Tables 2 and 3). During a subsequent phone follow-up, the patient did not follow discharge instructions to regularly review the coagulation function; however, the incision had largely healed at the most recent follow-up.

The patient experienced significant bleeding during cesarean section and heavy menstrual volume, which could not exclude previous coagulopathy-related diseases. There was no history of bleeding disorders among the immediate family members to rule out hereditary lesions. Laboratory findings demonstrated essentially normal liver function and a normal tumor series in women, excluding lesions of hepatic origin. FXIII level (20.2%), activated partial thromboplastin time (APTT), thrombin time (TT), and plasma thrombin time (PT) were normal. Fib was maintained at a low level (0.61–1.72 g/L), FDP, FM, and D-D were elevated, and the CI was < −3.0, in the thromboelastogram, in which the patient presented with a persistent bleeding state, leading to the assumption that it was due to secondary hyperfibrinolysis. In summary, persistent bleeding after surgery is thought to impair fibrin stabilization due to FXIIID in the low fibrinogen state, which is associated with secondary hyperfibrinolysis.

## 3. Discussion

This report summarizes a case of acquired FXIIID and secondary hyperfibrinolysis in a low fibrinogen state. In laboratory tests, the principal manifestations were decreased Fib and FXIII; normal APTT, TT, and PT; elevated FDP, FM, and D-D; and CI < −3.0. The clinical manifestations were dominated by persistent bleeding, which led to a diagnosis of acquired FXIIID and secondary hyperfibrinolysis. After treatment with Cryo, FFP, FC, and LPRC with hemostatic medications, the patient was discharged with improved coagulation and no significant bleeding from the incision.

Fibrin, a liver-derived plasma protein whose precursor is fibrinogen, is a key final component of the coagulation cascade and plays an important role in platelet aggregation and fibrin formation.^[1,2]^After surgery or trauma, vascular damage and thrombin action ultimately manifest as a state of rapid fibrinogen reduction, significant hypercoagulability, and hyperfibrinolysis, preventing clot expansion at the site of injury.^[3,4]^ Acquired hypofibrinogenemia is associated with hemodilution and excessive consumption of coagulation factors, and may be asymptomatic or hemorrhage/thrombosis similar to primary fibrin disorders.^[5–7]^ It manifests in patients with enhanced fibrinolysis as “delayed bleeding,” that is, bleeding manifestations several hours later due to enhanced fibrinolysis after initial hemostasis has been obtained after trauma.^[2]^ Preoperative fibrinogen deficiency in spine-related surgery was found to be significantly associated with perioperative bleeding in a prospective study.^[8]^ Abnormalities in fibrinogen can be initially suspected by prolongation of PT, APTT, etc, at which point specialized assays for the function and/or concentration of fibrinogen are required, such as the Clauss method, PT-based assays, and enzyme-linked immunosorbent assays. In patients with traumatic coagulopathy with severe bleeding and hypofibrinogenemia, treatment with FC or Cryo is recommended, and repeated dosing is required to maintain levels above 1.5 to 2 g/L because the patient is in an ongoing state of depletion.^[5,9–13]^ HGB supplementation is necessary to prevent anemia.^[14]^

Fibrinolysis is a complex physiological process that results in the breakdown of fibrin by plasma.^[13]^ Surgical stress, tissue destruction, and the release of inflammatory mediators lead to disruption of the physiological balance between coagulation and fibrinolysis.^[15–17]^ Excessive bleeding and hemodilution similarly induce abnormalities in coagulation and fibrinolytic function.^[18]^ Thrombosis can be attributed to postoperative hypercoagulability, blood stasis, and endothelial injury, whereas excessive bleeding is mostly due to secondary hyperfibrinolysis.^[19,20]^ Clinically, excessive bleeding manifestations are rarely related to hyperfibrinolysis because of the lack of a well-established gold standard for quantifying fibrinolytic function.^[2]^ Various laboratory tests can reflect fibrinolytic function, and measurement of fibrinolytic activity using biomarkers is currently the most accurate modality.^[21]^ Others, such as thromboelastograms, may also partially reflect fibrinolytic status but are not recommended for use alone to guide therapy.^[22]^ For the treatment of hyperfibrinolysis, the 2 most widely used antifibrinolytic drugs for the treatment of hyperfibrinolysis are TXA and epsilon aminocaproic acid,^[16,18]^ TXA is recommended within 1 hour after trauma for optimal clinical benefit.^[21]^

FXIII, also known as “fibrin stabilizing factor,” is distinct from other coagulation factors in that its potentially active subunit is present in a wide range of cells^[23,24]^ and is activated by thrombin and calcium ions (Ca^2+^) during the final stages of coagulation. Activated FXIII cross-links fibrin monomers and α2-plasmin inhibitors to fibrin, thereby increasing the clot resistance to mechanical stress and fibrinolysis.^[7,25–29]^ Excessive consumption of FXIII consumption due to trauma and surgery may increase the risk of postoperative bleeding.^[27,30]^ FXIII plays essential roles in hemostasis, angiogenesis, wound healing, bone regeneration, and inflammation.^[25,27,30–32]^ Patients with FXIIID may have physiologically normal results on routine coagulation tests; therefore, laboratory testing for FXIII activity is necessary in the presence of abnormal bleeding.^[13]^ In clinical practice, standardized activity tests based on transglutaminase activity, isopeptidase activity, direct quantitative antigen assays, coagulative resolution tests, or autoantibody assays are more feasible, and molecular genetics should be performed if necessary.^[30]^ Since the half-life of plasma FXIII is 11 to 14 days, it should be retested periodically. There is no uniform conclusion on the specific threshold of FXIII, and 60% to 70% is considered to be the critical value for the diagnosis of acquired FXIIID.^[30]^ The treatment with FXIIID mainly relies on alternative therapies, including cryotherapy, FFP, FXIII concentrates, and recombinant FXIII. However, FXIII concentrations vary with uncertainties in infusion timing and technical constraints; therefore, treatment modalities are not uniform.^[13,31,33]^ In previous case reports of abnormal bleeding, 1 male developed petechiae on the right arm and chest wall, and the other showed subcutaneous petechiae after reconstruction of the anterior cruciate ligament,^[33,34]^ both of whom were diagnosed with FXIIID and achieved satisfactory clinical results following cryo-based treatment.

## 4. Conclusion

We present a rare case in which we believe that impaired fibrin stabilization due to acquired FXIIID in the low-fibrinogen state and secondary hyperfibrinolysis caused this disease. Surgery should be performed with caution in patients with abnormal bleeding manifestations in the past, and the coagulation and fibrinolysis functions of the patient should be detected using a corresponding test to avoid unpredictable adverse outcomes due to misdiagnosis and missed diagnosis.

## Acknowledgments

The authors would like to express their gratitude to Cheng Jing, Professor at The Affiliated Hospitals of Shandong University of Traditional Chinese Medicine, for providing guidance on this article. We would like to thank our colleague Zhenguo Liu for helping with data collection.

## Author contributions

Supervision & Writing – review: Cheng Jing.

Writing – original draft & editing: Peng Zhang.

Visualization: Ruijing Zhang.
